# Territorial gaps on quality of causes of death statistics over the last forty years in Spain

**DOI:** 10.1186/s12889-023-17616-1

**Published:** 2024-02-03

**Authors:** Lluís Cirera, Rafael-María Bañón, Sergio Maeso, Puri Molina, Mónica Ballesta, María-Dolores Chirlaque, Diego Salmerón, Mortality Working Group of the Spanish Association of Epidemiology

**Affiliations:** 1 Department of Epidemiology, Regional Health Council, IMIB-Arrixaca. Ronda de Levante 11, 30008 Murcia, Spain; 2 Spanish Consortium for Research On Epidemiology and Public Health (CIBERESP), Calle de Melchor Fernández Almagro, 3, 28029 Madrid, Spain; 3 Department of Health and Social Sciences, University of Murcia, IMIB-Arrixaca, 32. 30120 Buenavista, Spain; 4 Medico-Legal Advisor. Ministry of Justice. Calle San Bernardo, 21. 28071 Madrid, Spain; 5 National Centre for Epidemiology, Carlos III Institute of Health (ISCIII), Avenida Monforte de Lemos 5, 28029 Madrid, Spain; 6 SGAIPE. Departament de Salut, Generalitat de Catalunya. Travessera de Les Corts, 131. 08028 Barcelona, Spain

**Keywords:** Mortality, Cause of death, Data accuracy, Reliability, Monitoring, Territoriality, Spain

## Abstract

**Background:**

The quality of the statistics on causes of death (CoD) does not present consolidated indicators in literature further than the coding group of ill-defined conditions of the International Classification of Diseases. Our objective was to assess the territorial quality of CoD by reliability of the official mortality statistics in Spain over the years 1980–2019.

**Methods:**

A descriptive epidemiological design of four decades (1980-, 1990-, 2000-, and 2010–2019) by region (18) and sex was implemented. The CoD cases, age-adjusted rates and ratios (to all-cause) were assigned by reliability to unspecific and ill-defined quality categories. The regional mortality rates were contrasted to the Spanish median by decade and sex by the Comparative Mortality Ratio (CMR) in a Bayesian perspective. Statistical significance was considered when the CMR did not contain the value 1 in the 95% credible intervals.

**Results:**

Unspecific, ill-defined, and all-cause rates by region and sex decreased over 1980–2019, although they scored higher in men than in women. The ratio of ill-defined CoD decreased in both sexes over these decades, but was still prominent in 4 regions. CMR of ill-defined CoD in both sexes exceeded the Spanish median in 3 regions in all decades. In the last decade, women’s CMR significantly exceeded in 5 regions for ill-defined and in 6 regions for unspecific CoD, while men’s CMR exceeded in 4 and 2 of the 18 regions, respectively on quality categories.

**Conclusions:**

The quality of mortality statistics of causes of death has increased over the 40 years in Spain in both sexes. Quality gaps still remain mostly in Southern regions. Authorities involved might consider to take action and upgrading regional and national death statistics, and developing a systematic medical post-grade training on death certification.

**Supplementary Information:**

The online version contains supplementary material available at 10.1186/s12889-023-17616-1.

## Introduction

The quality of statistics on causes of death (CoD) does not present consolidated indicators in papers nor in official health or statistical publications [1–5]. The quality of CoD statistics is assessed with several approaches and limited continuity. Only the great group of *symptoms, signs, and ill-defined conditions* of the International Classification of Diseases has achieved a generalised use [6–9]. This great quality group was often scored in wide ranges across nations when publicised, to suggest to take action [10]. The quality of statistics literature has also considered the impossible CoD, incompatible CoD to sex and age, and a variety of unspecific CoD; all referred as garbage medical causes [11–18].

Some collaborations have improved the reliability of ill-defined and garbage CoD by applying informatics algorithms to minimize the garbage codes in different ways: a) replacing the underlying CoD (which is the cause used in mortality statistics) with other causes filled out on the same death certificate; b) making expert data redistributions, or applying regression statistic models; or c) matching cases from health administrative databases for CoD extraction [11, 13, 15, 19–22].

Quality of death statistics have varied among countries for centuries. In the mid-eighteenth century, a little later than in North and Western European countries, Spain started to publish official vital statistics [23]. Recently, with the restoration of the Spanish democracy in 1978 and the decentralisation in the regions, a renewed boost was given to the improvement of civil registration and vital statistics to Western European standards [24]. Although the mortality rate decrease had begun time before, the amount of ill-defined causes of death has gradually decreasing throughout the new democratic time in Spain, to achieve, nowadays, an intermediate international position [10, 12].

The poor quality of CoD statistics involved a double miscertification issue concerning validity and reliability. Several validity studies showed a lack of uniformity across great and leading CoD, in Western context [25–29].

Our objective was to assess the national and regional quality of causes of death by analysing the reliability of the official mortality statistics over the years 1980–2019 across Spain.

## Methods

We implemented an observational descriptive epidemiological design over annual mortality cases by the administrative territorial division of Spain in 18 regions (named Autonomous Cities and Communities) (Supplementary Figure 1 Map). Every region supports the National Institute of Statistics (INE acronym in Spanish) by coding the CoD, except the cities Ceuta and Melilla where coding is carried out by the INE itself. We assessed reliability by 3 indicators. We grouped the underlying causes of death (CoD) into unspecific and ill-defined quality categories from the 9th and 10th revisions of the International Classification of Diseases. This assessment was performed by experts (three coding nurses, and epidemiologist and forensic physicians) and by taking into account the literature (Supplementary Annex A) [6, 12, 15]. Territorial case counts and populations of INE data are publicly available by an informed request (https://www.ine.es/infoine/?L=1). Time was tabulated in 4 decades (1980-, 1990-, 2000-, and 2010–2019). We estimated age-adjusted rates of quality categories for 100,000 inhabitants by using the direct method and the European Standard Population (WHO, 1976), as one indicator, and the weights {w_a_: a = 1,2,…,19} appear as Supplementary Table 1. For the second indicator, we presented the age-adjusted rates ratios (proportions, in percentage) of the quality category divided by its region all death causes. Both indicators, by decade and sex.

Each regional mortality rate was contrasted with the Spanish median of each decade by means of the Comparative Mortality Ratio (CMR) in a Bayesian framework, as the third indicator. The estimation was implemented as the number of deaths by region (${\text{r}}=1, 2, \dots , 18$) and age group (${\text{a}}=1 , 2, \dots , 19$), and it was modelled as a Poisson random variable ${{\text{d}}}_{{\text{ra}}} \sim \mathrm{ Poisson}({\upmu }_{{\text{ra}}})$, with the Jeffreys’s prior distribution [30] $\uppi ({\upmu }_{{\text{ra}}})\propto {{\upmu }_{{\text{ra}}}}^{-1/2}$. Again, we have used the European Standard Population for the age-adjusted rates. The mortality rate by region and age group is ${\uplambda }_{{\text{ra}}}={\upmu }_{{\text{ra}}}/{{\text{P}}}_{{\text{ra}}}$, where ${{\text{P}}}_{{\text{ra}}}$ is the population in the region ${\text{r}}$ and age group ${\text{a}}$. The mortality rate in Spain by age group is ${\uplambda }_{{\text{a}}}={\upmu }_{{\text{a}}}/{{\text{P}}}_{{\text{a}}}$, where ${\upmu }_{{\text{a}}}={\sum }_{{\text{r}}}{\upmu }_{{\text{ra}}}$ and ${{\text{P}}}_{{\text{a}}}={\sum }_{{\text{r}}}{{\text{P}}}_{{\text{ra}}}$. For each region, the adjusted rate is given by $\sum_{{\text{a}}}{{\text{w}}}_{{\text{a}}}{\uplambda }_{{\text{ra}}}$, and the comparative mortality rates ratio is the parameter:

$${{\text{CMR}}}_{{\text{r}}}=\frac{\sum_{{\text{a}}}{{\text{w}}}_{{\text{a}}}{\uplambda }_{{\text{ra}}}}{\sum_{{\text{a}}}{{\text{w}}}_{{\text{a}}}{\uplambda }_{{\text{a}}}}$$

The posterior distribution of each ${\upmu }_{{\text{ra}}}$ is a gamma distribution. The posterior distribution of the adjusted rate and of ${{\text{CMR}}}_{{\text{r}}}$ were obtained using the Monte Carlo method, generating ${\upmu }_{{\text{ra}}}^{{\text{i}}} \sim \mathrm{ Gamma}({{\text{d}}}_{{\text{ra}}}+1/2, 1)$ and computing ${\uplambda }_{{\text{ra}}}^{{\text{i}}}={\upmu }_{{\text{ra}}}^{{\text{i}}}/{{\text{P}}}_{{\text{ra}}}$, ${\uplambda }_{{\text{a}}}^{{\text{i}}}={\upmu }_{{\text{a}}}^{{\text{i}}}/{{\text{P}}}_{{\text{a}}}$, and ${{\text{CMR}}}_{{\text{r}}}^{{\text{i}}}=\sum_{{\text{a}}}{{\text{w}}}_{{\text{a}}}{\uplambda }_{{\text{ra}}}^{{\text{i}}}/\sum_{{\text{a}}}{{\text{w}}}_{{\text{a}}}{\uplambda }_{{\text{a}}}^{{\text{i}}}$, ${\text{i}}=1, \dots , 10 000$. The posterior estimation was performed using the median as the point estimator, and the 95% credible intervals (95% CrI) were the intervals from 0.025th to 0.975th quantile of the posterior distribution. The ${{\text{CMR}}}_{{\text{r}}}$ was considered statistically significant if the 95% CrI did not contain the value 1, to assess excess or defect on mortality. The analysis was performed using R version 3.6.1. The CMR function and the database are available on request.

## Results

### Spain by decade, sex, and quality category in rates indicator

**Fig. 1:**
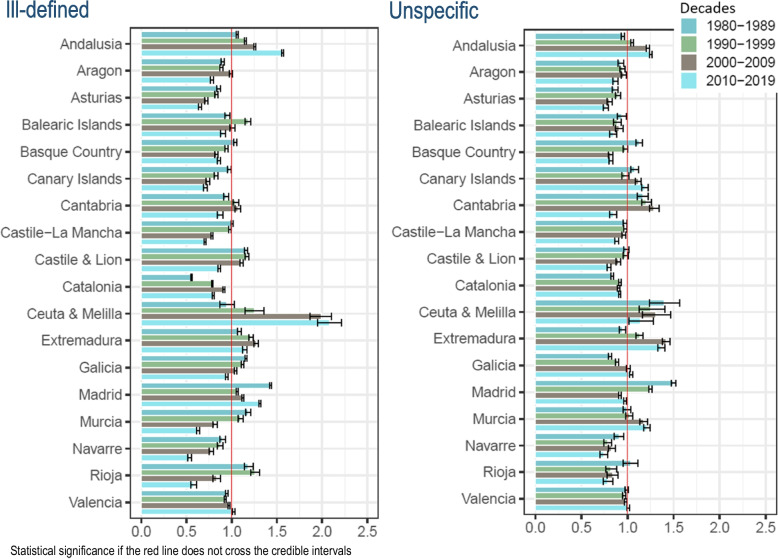
Regional comparative mortality ratios and 95% credible intervals of ill-defined and unspecific causes of death to Spain by decades. Both sexes, 1980–2019

All-cause deaths have decreased in in both sexes Spain as a whole over the years 1980 to 2019 (age-adjusted rates of 1 543 per 100,000 inhabitants in the first decade 1980–1989 to 867 per 100,000 inhabitants in the last decade of 2010–2019). The ill-defined CoD have declined in both sexes (from rates of 181, 101, 75, to 52 in the four decades studied) (Table and Fig. 1). The unspecific CoD have declined in both sexes, as well (from rates of 49, 39, 33, to 25 over decades) (Table 1, and cases distribution are available at Supplementary Table 2).

**Table 1:** Quality categories of causes of death rates^a^ by region, sex and decade. Spain, 1980–2019

		**Both sexes**					**Women**					**Men**				
		**1980–2019**	1980–1989	1990–1999	2000–2009	2010–2019	**1980–2019**	1980–1989	1990–1999	2000–2009	2010–2019	**1980–2019**	1980–1989	1990–1999	2000–2009	2010–2019
**Spain**	All	**1,098**	1,543	1,244	1,061	867	**877**	1,272	989	836	688	**1,393**	1,919	1,592	1,365	1,101
	Ill-defined	**84**	181	101	75	52	**79**	170	97	71	47	**88**	193	103	78	56
	Unspecific	**34**	49	39	33	25	**27**	41	31	27	20	**42**	60	51	41	31
Andalusia	All	**1,232**	1,717	1,390	1,204	982	**1,000**	1,426	1,121	968	792	**1,538**	2,129	1,756	1,519	1,224
	Ill-defined	**106**	191	116	95	81	**104**	184	114	93	79	**105**	198	115	93	81
	Unspecific	**38**	46	41	40	31	**32**	39	34	34	25	**47**	57	52	49	38
Aragon	All	**1,060**	1,430	1,167	1,015	850	**847**	1,197	938	799	665	**1,330**	1,725	1,459	1,293	1,084
	Ill-defined	**76**	163	89	75	40	**72**	156	88	71	37	**79**	170	89	78	43
	Unspecific	**32**	45	37	32	22	**27**	40	30	26	18	**38**	53	47	39	26
Asturias	All	**1,151**	1,584	1,281	1,092	910	**891**	1,280	987	836	704	**1,509**	2,006	1,696	1,451	1,194
	Ill-defined	**65**	154	84	54	33	**64**	148	83	55	32	**64**	161	82	49	34
	Unspecific	**29**	42	35	27	19	**23**	34	26	22	16	**36**	54	48	34	22
Balearic Islands	All	**1,147**	1,641	1,335	1,093	893	**920**	1,328	1,054	869	722	**1,438**	2,056	1,709	1,385	1,106
	Ill-defined	**85**	172	119	76	47	**79**	160	111	71	42	**90**	184	125	80	50
	Unspecific	**30**	46	35	30	21	**25**	38	27	26	18	**36**	56	46	35	25
Basque Country	All	**1,054**	1,526	1,204	1,014	824	**806**	1,187	917	769	635	**1,405**	2,035	1,625	1,368	1,088
	Ill-defined	**77**	187	95	63	44	**70**	167	86	59	41	**84**	216	105	65	47
	Unspecific	**31**	55	38	27	20	**23**	43	28	21	15	**41**	73	54	36	28
Canary Islands	All	**1,156**	1,675	1,345	1,155	918	**933**	1,386	1,067	919	744	**1,436**	2,048	1,710	1,457	1,128
	Ill-defined	**66**	176	84	56	37	**60**	167	79	48	32	**72**	184	87	63	42
	Unspecific	**36**	53	38	37	29	**28**	43	31	29	22	**45**	65	49	47	38
Cantabria	All	**1,074**	1,496	1,203	1,021	857	**825**	1,185	922	769	657	**1,420**	1,957	1,599	1,377	1,129
	Ill-defined	**81**	169	105	81	45	**74**	156	103	72	37	**86**	190	104	88	52
	Unspecific	**38**	57	47	42	21	**29**	44	35	33	17	**49**	77	65	56	26
Castile-La Mancha	All	**990**	1,345	1,086	941	794	**783**	1,110	864	729	619	**1,252**	1,647	1,367	1,211	1,012
	Ill-defined	**75**	181	99	59	36	**71**	173	95	56	34	**78**	191	101	61	38
	Unspecific	**32**	47	38	32	22	**27**	40	31	25	17	**40**	57	48	40	28
Castile & Lion	All	**1,084**	1,544	1,218	1,025	854	**898**	1,348	1,020	834	687	**1,309**	1,785	1,463	1,259	1,053
	Ill-defined	**90**	209	118	84	45	**88**	205	116	82	42	**91**	212	117	83	46
	Unspecific	**31**	48	38	30	20	**27**	44	32	26	18	**35**	54	47	34	22
Catalonia	All	**1,068**	1,465	1,217	1,041	846	**850**	1,211	962	817	668	**1,366**	1,826	1,577	1,350	1,084
	Ill-defined	**63**	100	79	69	41	**58**	94	74	63	37	**68**	108	84	74	46
	Unspecific	**30**	41	36	30	23	**24**	34	28	23	17	**39**	51	48	39	30
Ceuta & Melilla	All	**1,291**	1,787	1,390	1,257	1,045	**1,068**	1,450	1,133	1,040	875	**1,589**	2,336	1,757	1,553	1,256
	Ill-defined	**131**	171	126	150	107	**123**	155	118	146	99	**137**	192	136	148	115
	Unspecific	**43**	67	49	43	28	**35**	54	42	35	22	**54**	93	60	56	35
Extremadura	All	**1,172**	1,608	1,286	1,109	927	**941**	1,334	1,037	875	730	**1,467**	1,977	1,614	1,409	1,170
	Ill-defined	**101**	195	122	96	59	**97**	188	119	91	54	**103**	202	123	97	62
	Unspecific	**42**	46	44	47	34	**36**	40	37	40	28	**50**	55	55	55	41
Galicia	All	**1,086**	1,509	1,210	1,020	858	**861**	1,250	962	793	669	**1,387**	1,874	1,551	1,331	1,104
	Ill-defined	**92**	209	113	79	49	**86**	200	110	74	42	**96**	218	114	82	56
	Unspecific	**32**	39	35	33	26	**26**	34	27	26	21	**41**	47	46	43	32
Madrid	All	**977**	1,410	1,138	965	751	**772**	1,133	884	756	600	**1,277**	1,845	1,530	1,278	969
	Ill-defined	**103**	258	107	85	68	**90**	226	97	76	56	**118**	307	116	94	83
	Unspecific	**38**	73	49	30	24	**30**	58	38	25	19	**49**	98	67	38	32
Murcia	All	**1,180**	1,697	1,370	1,154	923	**968**	1,429	1,121	937	748	**1,452**	2,062	1,706	1,435	1,140
	Ill-defined	**76**	213	111	61	32	**77**	209	112	63	34	**71**	215	106	55	30
	Unspecific	**37**	48	40	39	30	**30**	40	33	32	24	**46**	60	50	48	37
Navarre	All	**1,011**	1,478	1,118	955	800	**786**	1,179	865	730	624	**1,312**	1,887	1,463	1,262	1,030
	Ill-defined	**64**	163	88	58	27	**60**	152	86	55	26	**67**	178	90	62	29
	Unspecific	**27**	44	31	27	18	**22**	37	25	23	14	**35**	55	40	34	24
Rioja	All	**1,043**	1,544	1,174	980	821	**819**	1,264	931	752	637	**1,330**	1,920	1,492	1,276	1,054
	Ill-defined	**78**	214	127	63	30	**76**	207	123	61	28	**80**	221	129	64	31
	Unspecific	**29**	50	32	28	19	**24**	45	26	24	16	**34**	57	40	32	24
Valencia	All	**1,168**	1,698	1,351	1,130	911	**950**	1,424	1,095	906	734	**1,452**	2,071	1,697	1,424	1,134
	Ill-defined	**80**	171	93	73	53	**78**	164	93	73	50	**79**	177	91	70	55
	Unspecific	**33**	48	38	32	25	**28**	42	31	27	20	**40**	58	49	39	31

^a^ Age-adjusted rates by the direct method to the Standard European Population per 100,000 inhabitants, expressed in rounded decimals to unit

Women and men rates have decreased over the decades in all quality categories. Men have showed higher rates than women have in all quality categories in Spain (Tables 2 and 3, and cases distribution are available at Supplementary Tables 3 and 4).

**Table 2:**
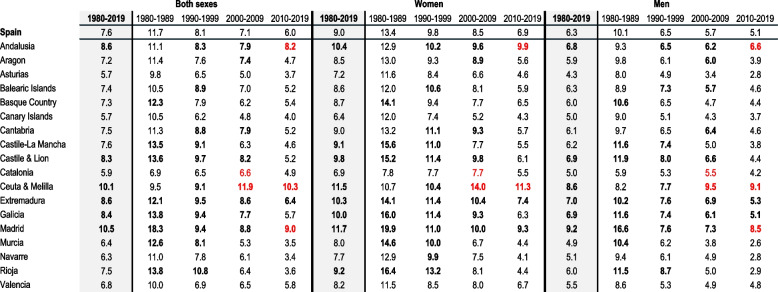
Proportions* of ill-defined causes of death by region, sex, and decade. Spain, 1980–2019

(*) Age-adjusted rates ratio (in %) in its all-causes mortality decade

Red colour (no bold) = Major or equal % than in one or more previous regional decades in the quality category

Bold (black or red) colour = Major or equal regional % than Spain in the same decade and quality category

**Table 3:**
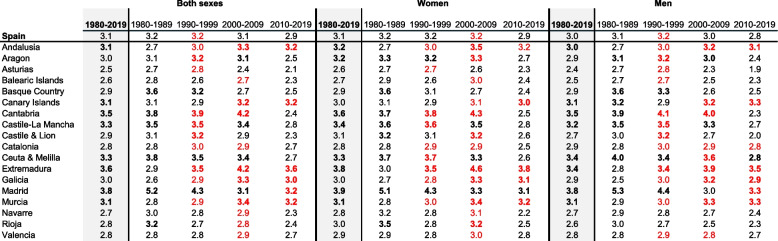
Proportions* of unspecific causes of death by region, sex, and decade. Spain, 1980–2019

(*) Age-adjusted rates ratio (in %) in its all-causes mortality decade

Red colour (no bold) = Major or equal % than any previous regional decades in the quality category

Bold (black or red) colour = Major or equal regional % to Spain in the same decade and quality category

In the whole period of 1980–2019, 8 of the 18 regions (that was, Andalusia, Asturias, Balearic and Canary Islands, Ceuta & Melilla, Extremadura, Murcia, and Valencia; with rate range from 1,147 to 1,291) have had higher all-cause age-adjusted rates than Spain (rate of 1,098) (Table 1).

Spain has achieved a 7.6% of ill-defined CoD in the whole period and both sexes. Ill-defined CoD were higher in women than men were (9.0 versus 6.3%). The proportion of Ill-defined CoD by sex has decreased over the decades, but is still prominent in women in all four decades (13.4, 9.8, 8.5, and 6.9%) (Table 2). In unspecific CoD, Spain has registered a 3.1% in the whole period and sex. This proportion has remained steady at rounded 3% over decades by sex (Table 3).

### Whole period, region and sex with rates indicator

In the whole period and in both sexes, seven of 18 regions (Ceuta & Melilla’s rate of 131, Andalusia 106, Madrid 103, Extremadura 101, Galicia 92, Castile & Lion 90, and Balearic Islands 85) have presented higher regional age-adjusted rates than Spain for ill-defined CoD. The same regions, excluding Balearic Islands, have displayed higher rates in women. Ill-defined rates were higher in men than women were in the whole period, with Murcia exception (rates of 71 versus 78, respectively). Men have showed higher rates in the same regions than in both sexes, plus Cantabria (Table 1).

All regions rates have decreased in all quality categories over time and sex. Five of the regions (Ceuta & Melilla rate of 107, Andalusia 81, Madrid 68, Extremadura 59, and Valencia 53) have exceed the Spanish rate in the last decade (2000–2019), for ill-defined CoD in both sexes. Women and men have showed the same pattern in the last decade but excluding Valencia in men (Table 1).

On unspecific CoD rates, 7 of 18 regions have exceed the Spanish rate in the last decade in both sexes (Extremadura 34, Andalusia 31, Murcia 30, Ceuta & Melilla 28, and Valencia 25). Women have pointed the same regions than have exceed in both sexes, while men have added one different more (Madrid), but excluding another (Valencia) (Table 1).

### Quality categories with proportions indicator

The regional ill-defined CoD have lowered in proportions through the most recent decades and sex, but three regions (Ceuta & Melilla, Andalusia, and Madrid) have maintained or increased proportions by sex, except in women in one region (Madrid), although it was higher than Spain (9 versus 7%) (Table 2). Regional unspecific CoD in both sexes have maintained the proportions over decades, meanwhile 6 of 18 regions have registered the lowest proportions in the last decade compared to Spain (Asturias, Balearic Islands, Cantabria, Castile & Lion, Navarre, and Rioja), and previous regional decades. Women have included 4 of 18 regions, while men have sex-specified this both sexes pattern, including one region (Aragon) (Table 3).

### Regional quality versus Spain by decade and sex with CMR indicator

The CMR of ill-defined CoD in both sexes have statistically exceeded Spain in 3 of the 18 regions (Andalusia, Extremadura, and Madrid) in all decades. The same excess has occurred in one other region (Ceuta & Melilla) in the last three decades. Another (Valencia) has registered excess mortality in the last decade (CMR = 1.02, 95% CrI 1.01 to 1.03). On the contrary, two regions (Castile & Lion and Galicia) that exceeded in the first three decades have decreased in the last (0.86, 0.85 to 0.88; and 0.95, 0.93 to 0.96, respectively) (Fig. 1 and Supplementary Table 5).

**Fig. 2:**
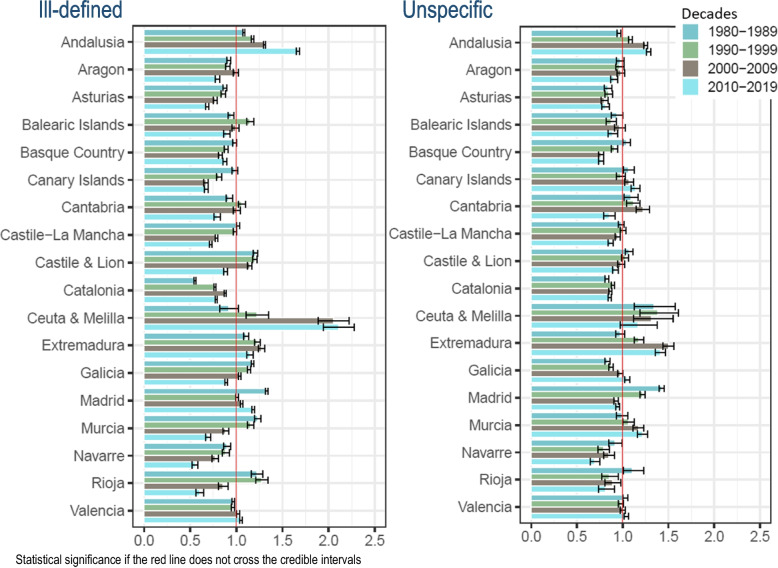
Regional comparative mortality ratios and 95% credible intervals of ill-defined and unspecific causes of death to Spain by decades. Women, 1980–2019

**Fig. 3:**
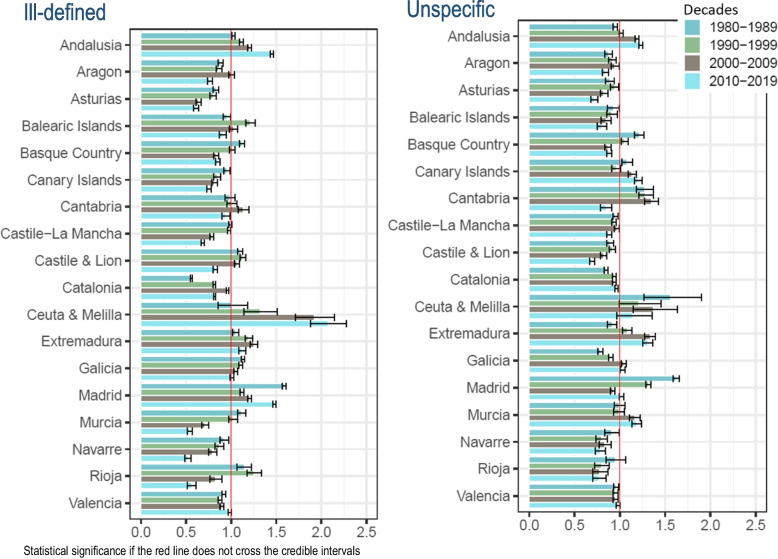
Regional comparative mortality ratios and 95% credible intervals of ill-defined and unspecific causes of death to Spain by decades. Men, 1980–2019

Women have showed an ill-defined mortality excess over Spain in 2 regions (Andalusia and Extremadura) in all decades; in one region (Ceuta & Melilla) in the last three decades; and in two different regions (Madrid and Valencia) in the last two decades (Fig. 2 and Supplementary Table 6). While, men have showed the same both sexes regional ill-defined pattern excess over all decades in the same 3 regions (Andalusia, Extremadura, and Madrid), and the same one in the last three decades (Ceuta & Melilla) (Fig. 3 and Supplementary Table 7).

The CMR of unspecific CoD in both sexes has statistically exceeded Spain in one region (Ceuta & Melilla) in all decades adding 5 more regions to the last decade (Andalusia, Canary Islands, Extremadura, Galicia, and Murcia) (Fig. 1 and Supplementary Table 8). Women and men have exceeded in 3 regions (Andalusia, Canary Islands, and Murcia) in in the last two decades; but also, Murcia and Extremadura in the last three decades, in women (Figs. 2 and 3, and Supplementary Tables 9 and 10, respectively).

## Discussion

The quality of mortality statistics of causes of death has increased throughout the 40 years studied in Spain in women and men. However, quality gaps still remain in specific regions. Meanwhile, the best regional quality results have showed that there is scope for targeted upgrade.

In our experience, two major components comprise reliable quality of CoD statistics: First component involves medical certification (professionalism, health record access, and healthcare administration type) and the second involves post-certification and related to mortality registers [31, 32], coding skills [33], and the capacity for documental health information recovery [12].

Our purpose was the internal comparison of the regions with Spain over a long period. The age-standardised rates to the European Standard Population fulfilled our purpose of national and international comparison. However, given the general rates decrease in the two quality of mortality categories, we chose to describe its proportion composition with respect to all-cause of deaths between year periods, thus giving a better description.

This study has some limitations. The CoD selection and quality grouping may lack of comparability. However, our consensus on ICD10 code selection was based on the ICD10 instructions manual [34] and literature revision [6, 12, 35]. The two major proposals of quality assessment of CoD come from the Centers for Disease Control [6] and Anaconda software® [35], but these showed some qualitative differences. The CDC paper established 3 subtypes of CoD (unknown & ill-defined, immediate & intermediate, and nonspecific). The unknown and ill-defined causes included fewer codes than the ICD10 (18th chapter and annex 7.3). These immediate or intermediate CoD could also be reassigned to a general unspecific group, as well as to the ill-defined group by WHO criteria (ICD10 code I50 for heart failure). The annual national summary of 2.2% for unknown and ill-defined causes versus a 32.5% for the other unsuitable CoD, seems a broad gap to take action (Supplementary Annex B) [6, 12]. The Anaconda software encompasses 3 axes: the 1st axe, five qualify for uninformative subtypes (1- symptoms, sign, and ill-defined conditions; 2- impossible as CoD; 3- intermediate CoD; 4- immediate CoD; and 5- insufficiently specified CoD extracted from Global Burden Disease (GBD) [36]; the 2nd axe, four levels of health impact policies of 800 codes (Supplementary Annex B); and the 3rd axe, a vital performance index (of completeness, and garbage and impossible codes by age and sex). Although, GBD is dynamically updated [37], this praiseworthy effort, also expresses complex assessment outcomes, to take action further than lack of completeness [27, 38] and high numbers for ill-defined CoD, especially in low-income countries [10, 39]. In our case, for example, we considered dementia, ictus, pneumonia, or accidental poisoning by narcotics, reliable as primary health care diagnostics, however a detailed hospital-like testing technology may improve their accuracy.

The health and judicial administration framework matters in medical certification. Spain is supported by a Welfare State with National Health and Social Systems (public funding, universal access, a majority of centres of governmental propriety, and regional competences in health and social care budget and management), as well as a judicial system with forensic pathology and laboratory facilities at every regional centre. The Western European context of public funding and universal health care provision (private versus governmental) could be associated with completeness and validity of causes of death [40]. Likewise, the majority of diseases can be diagnosed through anamnesis and conventional physical examination and complementary tests at the Primary Health Care subsystem.

The process of completing and accurately coding a death certificate according to the ICD is challenging for all countries. Not all of them have achieved a good-quality threshold on mortality data. The WHO included in the medium-quality category several high-income Western European countries (such as Austria, Belgium, Denmark, France and Germany, regulated by universal Health Insurance systems) [14, 41]. Furthermore, in the present decade six high-income countries worldwide achieved a 9—31%, adding ill-defined (ICD10, 18th chapter) to impossible CoD [15]. Similarly, it would be a specificity error to classify 67.3% of the vital registration deaths as least-specific codes, without any further geographical or social context reference [21]. Providing the magnitude of poor quality death certification, health authorities did not seem to play a role in the probable random underestimation of the great and leading CoD [7, 13, 26–28, 31]. Currently, the COVID-19 pandemic has probably worsened death certification [42]. Statistics and health authorities may consider implementing the framework conditions to avoid miscertification. In addition, the WHO may include ill-defined conditions in the same ICD chapter in future revisions. Meanwhile, some national CoD registries have achieved top quality [32], and could be a standard target to replicate.

Some papers have emphasised the weakness of imputations made by case identification algorithms based on available health and population record information. The imputations from multiple search assignments of unsuitable CoD were proportionally predicted [11, 13, 15, 19–21, 43, 44] without a representative sample of validation [7, 13, 27, 28, 31].

Related to sex differences, we have assessed lower quality death certification in men than women at any territory and decade. Results, that are aligned to multi-country study that stated a no clear bias against women in death registration [45]. The three indicators applied adjusted for age groups. Sex differential in longer life might partially contributed to major medical unspecificity by polipathology bias. The general (not much) higher proportion quality in women than men on bad mortality quality, should consider that the magnitude of the population involved is described by the rates, which were higher in men than in women.

The poor statistical death quality showed in regions is much coincident to regional distribution of the Gross Domestic Product per Capita by regions of Spain (Supplementary Figure 2 Map). Moreover, poor death certification may be linked to individual characteristics (such as medical professionalism, the social stratum of the deceased, etc.) [46, 47]. This misclassification would imply regional and individual differential errors. The long-time evidence of our results is suggestive of a new organisational model with a multilevel health experts support to the National Institute of Statistics for a better regional and national standards upgraded [33].

Some studies have associated the deficiency of medical specialist education on death certification with miscertification in mortality statistics [48]. Courses for the improvement of CoD notification have oftentimes been imparted with diverse approaches and to different alumni, such as medical students or physicians in their specialisation [43, 49, 50]. Nowadays, this training is available through new communication technologies such as mobile phones [44], websites and e-learning platforms [43, 51]. Additionally, the WHO may introduce a certification of “medical competence on certification of causes of death” to foster the quality of mortality statistics worldwide.

As stated before, there is a general need of representative national validity studies of causes of death to address properly the post-certification informatics reassignment in CoD.

## Conclusion

The reliability of the CoD has been improving over the last 40 years in Spain in both sexes. Regional gaps have persisted along those years and even in the last decade. Regional gaps mostly focused in Southern regions. Authorities involved might consider to take action and upgrade bad quality, and to develop a systematic medical post-grade training on death certification to improve regional differences and the quality of death statistics of Spain.

### Supplementary Information

**Additional file 1: Supplementary Annex A.**

**Additional file 2: Supplementary Annex B.**

**Additional file 3: Supplementary Tables 1-10.**

**Additional file 4: Supplementary Figure 1 Map.**

**Additional file 5: Supplementary Figure 2 Map.**

## Data Availability

The dataset and the analysis R-script file used during the current study are available from the corresponding author.
