# Supplementary material for: Territorial gaps on quality of causes of death statistics over the last forty years in Spain
Source: BMC Public Health. 2024 Feb 3;24:361. doi: 10.1186/s12889-023-17616-1 (PMC10837971; doi:10.1186/s12889-023-17616-1)
Supplement: Supplementary file 1 — Additional file 1: Supplementary Annex A. [file 12889_2023_17616_MOESM1_ESM.docx]

**Supplementary Annex A. Definitions of the quality groups* of causes of death and their codes** according to the 9^th^ and 10^th^ revisions of the International Classification of Diseases (ICD).**

**Definitions**

**Ill-defined causes** = All of the codes from chapter XVI of the ICD-9 (Symptoms, Signs, and Ill-Defined Conditions), besides chapter XVIII of ICD-10 (*Symptoms, signs and abnormal clinical and laboratory findings, not elsewhere classified*), and ill-defined conditions list of the *instruction manual* of ICD-10 (p. 213), and other codes included by the Spaniard consensus.

**Unspecific causes** = Codes with four character “.9”, which do not indicate the organ affected, but instead provided only the affected system, in addition to other causes applied without sufficient information, or other external causes which were not specified to a cause or manner of death.

**Codes**

**Ill-defined causes**

- ICD9: 4275, 4289, 4589, 4590, 765, 768, 7799, 780-799 (except 7958).

- ICD10: I46, I509, I959, I99, J960, J969, P07, P20, P21, P285, P95, P969, R00-R74, R76-R94, R96-R99.

**Unspecific causes**

- ICD9: 1369, 1398, 1499, 1599, 1659, 1849, 1879, 1899, 1929, 195, 199, 2119, 2129, 2219, 2299, 2239, 2249, 2309, 2298, 2259, 2279, 2319, 2333, 2336, 2339, 2349, 2389, 2390, 2391, 2599, 2779, 2799, 2899, 3159, 3481, 3499, 3799, 3889 4599, 5089, 514, 5199, 5999, 6089, 6259, 6299, 6469, 6559, 6569, 6599, 6749, 7042, 7079, 7099, 7189, 7399, 7609, 7619, 7639, 7709, 7779, 7798, E9889.

- ICD10: B89, B949, B99, C269, C399, C579, C639, C689, C729, C76, C80, C969, D019, D024, D073, D076, D091, D099, D139, D144, D159, D289, D299, D309, D339, D359, D369, D379, D386, D409, D419, D439, D489, D759, D899, E349, E639, E649, E889, F89, F99, G931, G969, G98, H579, H939, I879, J709, J81, J989, L29, L679, L89, L989, M249, N179, N399, N509, N949, O269, O359, O369, O759, O909, O95, O96, O97, P009, P019, P039, P289, P299, P789, P968, Y34.

-------------

* Sorted by the first three characters and for the fourth one if ICD code was available.

** Three character codes include all fourth possible ones.

Own elaboration.

Source: Mortality Working Group, Spanish Society of Epidemiology.
