# Supplementary material for: Territorial gaps on quality of causes of death statistics over the last forty years in Spain
Source: BMC Public Health. 2024 Feb 3;24:361. doi: 10.1186/s12889-023-17616-1 (PMC10837971; doi:10.1186/s12889-023-17616-1)
Supplement: Supplementary file 2 — Additional file 2: Supplementary Annex B. [file 12889_2023_17616_MOESM2_ESM.docx]

**Supplementary Annex B. Unreliable code counts up to 3^th^ - character of the International Classification of Diseases - 10^th^ Revision**

| Global Burden Disease ^*^ | |
| --- | --- |
| Garbage codes with serious policy implications – Level 1.  The most difficult to redistribute a CoD into the 3 major groups (communicable and non-communicable diseases, and Injuries). | 2 315 |
| Garbage codes with substantial implications – Level 2 | 350 |
| Garbage codes with important implications - Level 3 | 382 |
| Garbage codes with limited implications - Level 4 | 68 |
| Total | 800 |
|  |  |
| National Vital Statistics Reports - CDC, USA ^/^ | |
| Unknown and Ill-defined causes of death | 101 |
| Immediate and Intermediate causes of death | 108 |
| Nonspecific CoD | 168 |
| Total | 377 |
| Spanish Society of Epidemiology (Annex 1) | |
| Ill-Defined CoD | 109 |
| Nonspecific CoD | 75 |
| Total | 184 |

CoD = Underlying Cause of Death

(*) In Supplementary appendix 1. GBD 2017 Causes of Death Collaborators. Global, regional, and national age-sex-specific mortality for 282 causes of death in 195 countries and territories, 1980–2017: a systematic analysis for the Global Burden of Disease Study 2017. Lancet 2018; 392: 1736–1788. p. 407.

**Source:** Own elaboration
