# Supplementary figures and images for: Territorial gaps on quality of causes of death statistics over the last forty years in Spain
Source: BMC Public Health. 2024 Feb 3;24:361. doi: 10.1186/s12889-023-17616-1 (PMC10837971; doi:10.1186/s12889-023-17616-1)

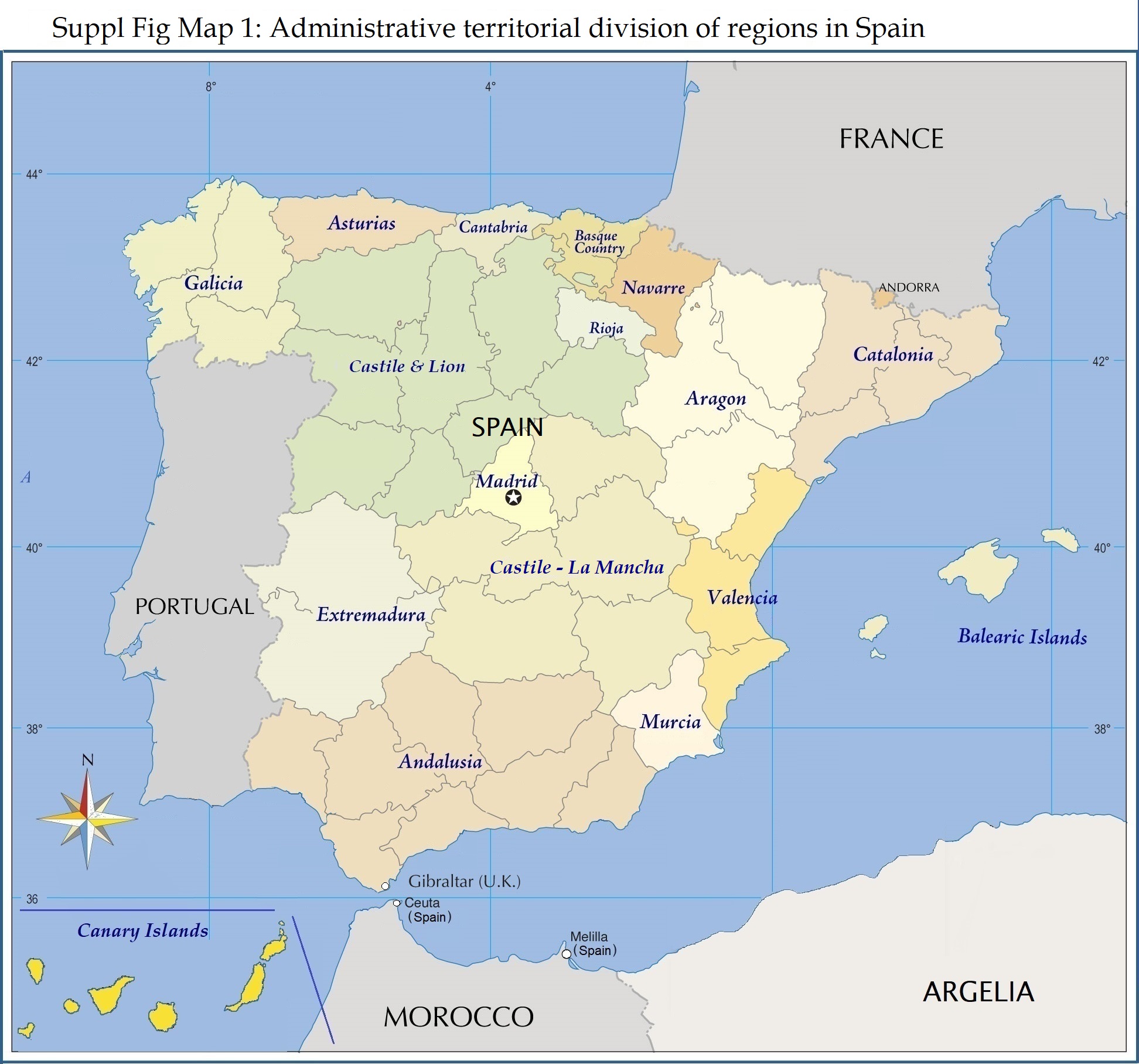

Supplement: Supplementary file 4 — Additional file 4: Supplementary Figure 1 Map. [file 12889_2023_17616_MOESM4_ESM.jpg]

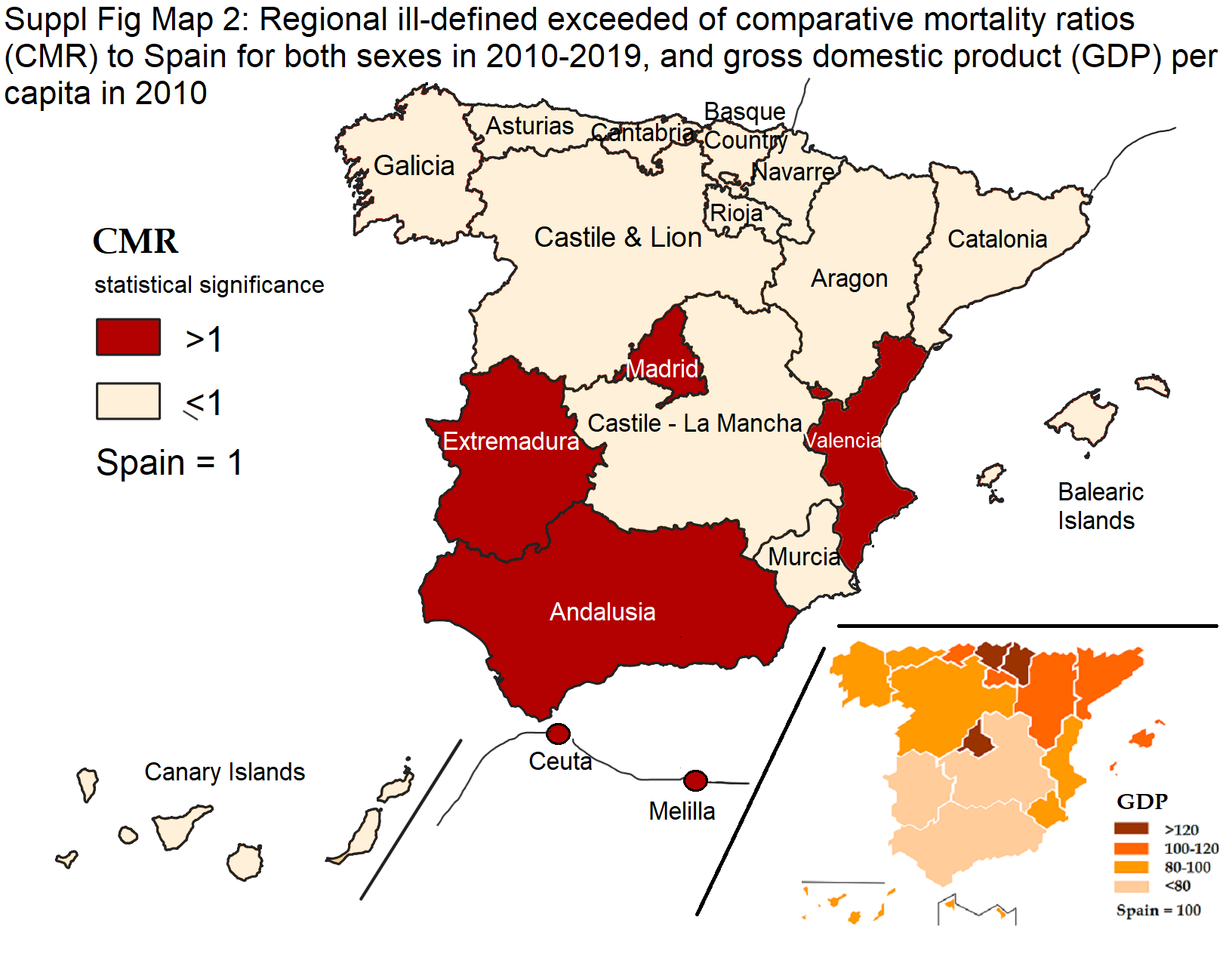

Supplement: Supplementary file 5 — Additional file 5: Supplementary Figure 2 Map. [file 12889_2023_17616_MOESM5_ESM.png]
